# Detecting significant single-nucleotide polymorphisms in a rheumatoid arthritis study using random forests

**DOI:** 10.1186/1753-6561-3-s7-s69

**Published:** 2009-12-15

**Authors:** Minghui Wang, Xiang Chen, Meizhuo Zhang, Wensheng Zhu, Kelly Cho, Heping Zhang

**Affiliations:** 1 Department of Epidemiology and Public Health, 60 College Street, Yale University School of Medicine, New Haven, Connecticut 06520, USA

## Abstract

Random forest is an efficient approach for investigating not only the effects of individual markers on a trait but also the effect of the interactions among the markers in genetic association studies. This approach is especially appealing for the analysis of genome-wide data, such as those obtained from gene expression/single-nucleotide polymorphism (SNP) array experiments in which the number of candidate genes/SNPs is vast. We applied this approach to the Genetic Analysis Workshop 16 Problem 1 data to identify SNPs that contribute to rheumatoid arthritis. The random forest computed a raw importance score for each SNP marker, where higher importance score suggests higher level of association between the marker and the trait. The significance level of the association was determined empirically by repeatedly reapplying the random forest on randomly generated data under the null hypothesis that no association exists between the markers and the trait. Using random forest, we were able to identify 228 significant SNPs (at the genome-wide significant level of 0.05) across the whole genome, over two-thirds of which are located on chromosome 6, especially clustered in the region of 6p21 containing the human leukocyte antigen (HLA) genes, such as gene *HLA-DRB1 *and *HLA-DRA*. Further analysis of this region indicates a strong association to the rheumatoid arthritis status.

## Background

Rheumatoid arthritis (RA), a common autoimmune disease with pathological symptom of swelling and inflammation, may cause severe damage to diarthrodial joints and even movement disability [1]. Approximately 1% of the adult populations in the world are affected by RA, and women are more vulnerable to this disease than men [2]. Research has led to improvements in RA therapy, but the etiology of the pathophysiological and molecular mechanisms underlying RA is not well understood.

Random forest has the ability to investigate the effect of the interactions among the markers in genetic association studies. A random forest consists of many classification trees and is known to be more reliability than a single tree in classification. Thus, it has been widely used for gene expression and single-nucleotide polymorphism (SNP) data analyses [3-5]. Using a random forest, one can estimate the impact of each variable on prediction results by calculating a measurement called importance [6]. For example, Sun et al. used random forest for the RA status prediction on the Genetic Analysis Workshop (GAW) 15 RA data including 5742 SNPs [3]. Although no single SNP was significant, they achieved an appealing prediction performance with their top 500 SNPs ranked by importance index. Based on these results, Sun et al. were able to accurately predict RA patients with high sensitivity and specificity. This conclusion can be explained by the fact an importance score is based not only on the contribution of a single variable, but also on its interactions with other variables. The importance index is a better measurement of an "overall" association of the variable with the disease compared to other statistics, e.g., the allelic chi-square statistic, designed for single marker association tests. However, one shortcoming of the importance index is that it does not seem to have an apparent, simple asymptotic distribution. Thus, it is hard to find significant SNPs from the results of importance index, even when we know high-ranking SNPs should have a better chance of being significant than low-ranking SNPs. Similar approaches to identifying important genes have been proposed by Chen et al. [4] for genome-wide studies and by Rodenburg et al. [7] for microarray data. In this study, we address this issue by estimating empirical *p*-values for importance index using the North American Rheumatoid Arthritis Consortium (NARAC) SNP data. We also apply a permutation model with the null hypothesis of no association between SNPs and RA status. Using a random forest approach, we were able to detect SNPs with significant importance.

## Methods

### Dataset

The GAW16 Problem 1 RA dataset comprises whole-genome data from NARAC. It contains 868 cases and 1,194 controls, and 545,080 SNP-genotype fields from the Illumina 550 k chip. In this work, we first consider all 531,689 autosomal SNPs, discarding the mitochondrial, X, Y, and pseudo-autosomal chromosomes. By removing those SNPs with excessive missing data (missing >20%), we analyzed a total of 530,959 autosomal SNPs.

### Random forest

Random forest is a combinational classifier proposed by Breiman [6] that includes multiple individual classification trees. A classification tree is a classifier that uses a graphic tree model. The prediction procedure is based on a recursive partitioning method [8]. Compared to a single classification tree, a random forest is regarded as a more reliable and robust alternative. To build a random tree in the random forest, first, a bootstrap dataset is formed by sampling with replacement from a training set. The size of the bootstrap dataset is equal to the training dataset. The unsampled data is known as the out-of-bag data. Next, a random set of the variables at each node is selected, and then the tree is fully grown without pruning. Finally, the previous process is repeated to develop multiple individual random trees. The strength of random forest lies in the fact that the ensemble can achieve both low-bias and more accurate results than a single classification tree.

An important feature of the random forest is the variable importance index [6], reflecting the contribution of a variable to the improvement of classification. Specifically, after a tree *t *is built from a bootstrap dataset, the out-of-bag samples are used as its test set. The number of correctly classified samples, *count*_*t*, *ini*_, is calculated. Next, for each variable *v*, the values of variable *v *in the out-of-bag samples are randomly permuted. This permuted dataset is used as the tree *t*'s new test set, and number of correctly classified samples, *count*_*t*, *v*_, based on this permuted dataset is calculated. Finally, the raw importance score for the variable *v *is defined as:

where *N *represents the number of trees in random forest.

### Test procedure

The test procedure proceeds in three steps. First, we apply the random forest by using individual SNPs as features and the case/control disease status as outcome and calculate the raw importance score of each SNP. Here the number of trees used in random forests is set to 20,000. Because of the massive number of SNPs (530,959), the number of SNPs used to split on at each node is set to be  = 728. So statistically every SNP can be sampled at each node, especially at the root node. Second, to evaluate the significance of raw importance scores for a single SNP in its association with the disease status, the status of case/control is permuted randomly in the whole dataset. Then we reapply the random forest method on the permuted dataset and recalculate the importance scores. The maximum importance score over all the SNPs is recorded. Finally, the second step is repeated 5,000 times. Under the null hypothesis of no association between SNPs and the disease, a distribution of the maximum importance of single SNP is obtained based on the 5,000 runs, which can be used to assess the significance level of raw importance score in the original dataset.

## Results

At the genome-wide significance level of 0.05, we found 228 significant SNPs across the whole genome, and the number of the significant SNPs in each chromosome is shown in Table 1. Chromosome 6 has the largest number of significant SNPs associated with the RA status (181 SNPs), while chromosomes 19 and 9 have nine and eight significant SNPs, respectively. All three of these chromosomes have been reported to be in linkage with the RA disease locus [9-15].

**Table 1:** Numbers of significant SNPs in 22 autosomal chromosomes

Chromosome	No. SNPs
1	3
2	0
3	1
4	0
5	1
6	181
7	2
8	2
9	9
10	1
11	2
12	3
13	1
14	5
15	1
16	2
17	2
18	0
19	8
20	1
21	0
22	3

**Figure 1:**
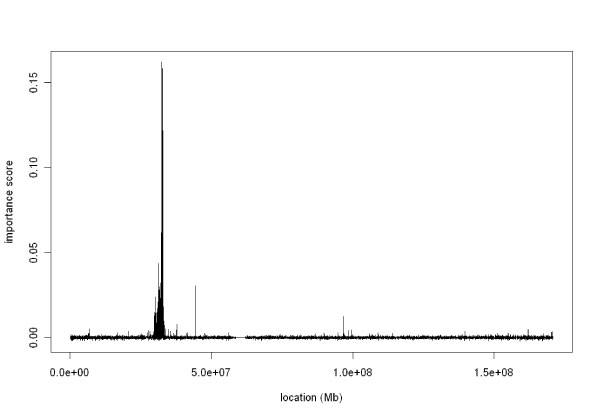
**The raw importance scores of SNPs on chromosome 6**.

Figure 1 further illustrates the details of the results on chromosome 6. Almost all significant SNPs (178 out of 181) are clustered into a small region, which we later found to be a part of HLA region in 6p21. Further analysis revealed that this region contains many known RA related genes, such as *HLA-DRB1 *and *HLA-DRA1 *[9,10]. Moreover, these 178 significant SNPs are related to known genes according to the NCBI SNP database. For example, SNPs with highest importance score (rs3129871, rs3129882, rs2239804, and rs7192) are all located in the ORF of *HLA-DRA1*, the HLA class II alpha chain paralogs, which works with *HLA-DRB1 *to form the class II heterodimer by anchoring to the cell membrane. It is noteworthy that there are five significant SNPs (rs3817973, rs2076530, rs3817963, rs3793126, and rs3806156) belonging to gene *BTNL2*, which was previously identified as predisposing to the RA diseases [11]. This association may be due to the strong linkage disequilibrium with *HLA DQB1-DRB1 *haplotypes [12].

Tables 2 and 3 list the significant SNPs on chromosomes 9 and 19. The names of genes and the chromosomal positions where those SNPs reside are also given. Most of the significant SNPs are located in the 9q33-34 and 19p13 regions. Several SNPs in the 9q33.2 region have been identified to be associated with RA [14]. In addition, we identified a few novel regions, such as 9q24.3, to be associated with RA. On chromosome 19, Thompson et al. [15] detected a significant linkage peak in the 19p13 region for juvenile RA; however, to our knowledge, no association studies have reported findings in this region. The genes displayed in Tables 2 and 3 have not been previously found to be associated with RA, and hence warrant further investigation.

**Table 2:** Significant SNPs in chromosomes 9

SNP	Gene	Location
rs636922	*C9orf66*	9p24.3
rs2148843^a^	-	-
rs3124237	*DAPK1*	9q34.1
rs913935	*MORN5*	9q33.2
rs2235056	*LMX1B*	9q34
rs2282010	*UCK1*	9q34.13
rs496503	*KCNT1*	9q34.3
rs9411216	*AGPAT2*	9q34.3
rs3812499	*PNPLA7*	9q34.3

^a^There is no known gene associated with the SNP.

**Table 3:** Significant SNPs in chromosomes 19

SNP	Gene	Location
rs349313	*ARID3A*	19p13.3
rs4147918	*ABCA7*	19p13.3
rs313784	*BRUNOL5*	19p13
rs7250947	*KIAA1881*	19p13.3
rs646816^a^	-	-
rs2216670	*MED26*	19p13.11
rs732841^a^	-	-
rs10423006	*GLTSCR1*	19q13.3

^a^There is no known gene associated with the SNP.

## Conclusion

In this paper, we adopted the random forest approach for RA study on GAW16 Problem 1 and used a permutation procedure to estimate the significant level. As previously reported, our analysis confirmed that the region in 6p21 of chromosome 6 contains an abundance of significant SNPs. Also we found that some of the significant SNPs are related to known genes such as *HLA-DRA1 *and *BTNL2*. Moreover, we also detected two regions on chromosomes 9 and 19 for which significant linkage signals were previously reported. These results demonstrate that random forest can be a useful tool in detecting markers and chromosome regions that are in linkage disequilibrium with the disease alleles.

## List of abbreviations used

GAW: Genetic Analysis Workshop; NARAC: North American Rheumatoid Arthritis Consortium; RA: Rheumatoid arthritis; SNP: Single-nucleotide polymorphism

## Competing interests

The authors declare that they have no competing interests.

## Authors' contributions

MW and HZ designed the study, carried out the data analysis, and drafted the manuscript. XC, MZ, and WZ participated in data analysis. KC helped to draft the manuscript. All authors read and approved the final manuscript.
